# Diffuse pulmonary arteriovenous malformation presenting with secondary polycythemia and headaches: a case report

**DOI:** 10.1186/s13256-024-04643-8

**Published:** 2024-07-08

**Authors:** Salaar Ahmed, Amna Irfan Ansari, Abdullah Saeed Khan, Javaid Ahmed Khan

**Affiliations:** 1 Medical College, Aga Khan University, Stadium Road, Karachi, 74800 Pakistan; 2 Department of Medicine, Aga Khan University Hospital, Karachi, Pakistan

**Keywords:** Pulmonary arteriovenous malformations, AVMs, HHT, Headache, Polycythemia

## Abstract

**Background:**

Pulmonary arteriovenous malformations are a relatively uncommon medical condition, affecting roughly 1 in every 2500 individuals. Of those suffering from pulmonary arteriovenous malformations, 80% have an underlying genetic condition: hereditary hemorrhagic telangiectasia.

**Case presentation:**

We present the case of a 20-year-old Pakistani male with a history of persistent slower-onset frontal headaches that increased in severity within the course of the day. His hemoglobin was 18 g/dl, indicating polycythemia, for which he had undergone seven venesections in a month previously. His physical examination was unremarkable. His computed tomography scan depicted multiple dilated tortuous vessels with branching linear opacities in the right lower lobe of the lungs. The multiple feeding arteries were supplied by the right main pulmonary artery, and the large draining veins led to the right inferior pulmonary vein. This was identified as a diffuse pulmonary arteriovenous malformation. He was recommended for a right pulmonary artery angiogram. It showed multiple tortuous vessels with a nidus and large draining veins—features of a diffuse arteriovenous malformation in the right lower lobe of the lung consistent with the computed tomography scan. Embolization of two of these vessels feeding the arteriovenous malformation was conducted, using Amplatzer Vascular plug 2, whereas multiple pushable coils (five coils) were used for embolizing the third feeding vessel. This achieved 70–80% successful embolization of right pulmonary AVM; however, some residual flow was still seen in the arteriovenous malformation given the complexity of the lesion. Immediately after, his oxygen saturation improved from 78% to 96%.

**Conclusion:**

Diffuse pulmonary arteriovenous malformations, as seen in this patient, are rare, accounting for less than 5% of total pulmonary arteriovenous malformations diagnosed. The patient presented with a complaint of progressive frontal headaches, which can be attributed to low oxygen saturation or the presence of a cerebral arteriovenous malformation. There was no history of hereditary hemorrhagic telangiectasia in the patient’s family. Furthermore, although most patients with hereditary hemorrhagic telangiectasia and hence pulmonary arteriovenous malformation have complaints of iron-deficiency anemia, our patient in contrast was suffering from polycythemia. This can be explained as a compensatory mechanism in hypoxemic conditions. Moreover, the patient had no complaint of hemoptysis or epistaxis, giving a varied presentation in comparison with a typical pulmonary arteriovenous malformation.

## Background

Pulmonary arteriovenous malformations (PAVMs) are aberrant connections between the pulmonary artery and vein. The majority of PAVMs are present in patients with hereditary hemorrhagic telangiectasia (HHT), a genetic disorder [1–3]. PAVM is a relatively uncommon medical condition and presents with dyspnea on exertion, cyanosis, and hemoptysis [4]. Diagnosis is mostly based on clinical suspicion followed by a CT angiogram [5]. They are classified as simple, complex, and diffuse, with simple being more common [6, 7]. The principle for treating PAVM is obliteration of the abnormal vasculature via an open or endovascular approach, with the latter depending on vascular plugs [8]. Our study aims to present the case of a patient with a chief complaint of headache who was ultimately diagnosed and treated along the lines of PAVM.

## Case presentation

A 20-year-old Pakistani male college student from Balochistan developed complaints of slow-onset frontal headache for 1 month. This headache persisted and increased in severity throughout the day and decreased with analgesics. Initial investigations revealed hemoglobin of 18 g/dl (normal range in males: 12.3–16.6). He was subsequently managed on the lines of primary idiopathic polycythemia, which led to seven venesections in 1 month, with 500 ml of blood being removed each time. He had also been given 75 mg of aspirin once daily for 1 month. However, the symptoms did not resolve, owing to which a chest X-ray and computed tomography (CT) scan were ordered to rule out secondary polycythemia.

The CT, which was performed at a different center (owing to cost), revealed branching linear opacities in the right lower lobe with an AVM of the right lower lobe, a dilated right main pulmonary artery, and a dilated right inferior pulmonary vein. He then presented to our hospital for further management. His physical examination did not reveal any significant findings. He did not appear in any visible respiratory distress. There was no cyanosis, clubbing, or any visible telangiectasias. He denied dyspnea on exertion and hemoptysis. He also did not have any history of epistaxis or blood in stools. He was afebrile, and vitally stable with a blood pressure (BP) of 129/70 mmHg, a respiratory rate of 22, but a resting oxygen (O_2_) saturation of 82%, for which he was put on supplemental oxygen. Spirometry revealed a moderately restrictive pattern, with a forced expiratory volume in the first second (FEV1) of 89% and a ratio of the forced expiratory volume in the first second to the forced vital capacity of the lungs (FEVI/FVC) of 92%. Diffusion lung capacity (DLCO) was not ordered. He was electively admitted for percutaneous transcatheter angioembolization of the PAVMs.

A right femoral venous approach was used. Initially, a right pulmonary artery angiogram was performed, which showed multiple tortuous vessels with a nidus and large draining veins consistent with a large diffuse AVM in the right lower lobe. This was consistent with the findings of the CT scan. It was supplied mainly by the right lower lobe branch of the pulmonary artery, and multiple veins were draining the AVM into the left atrium.

**Fig. 1:**
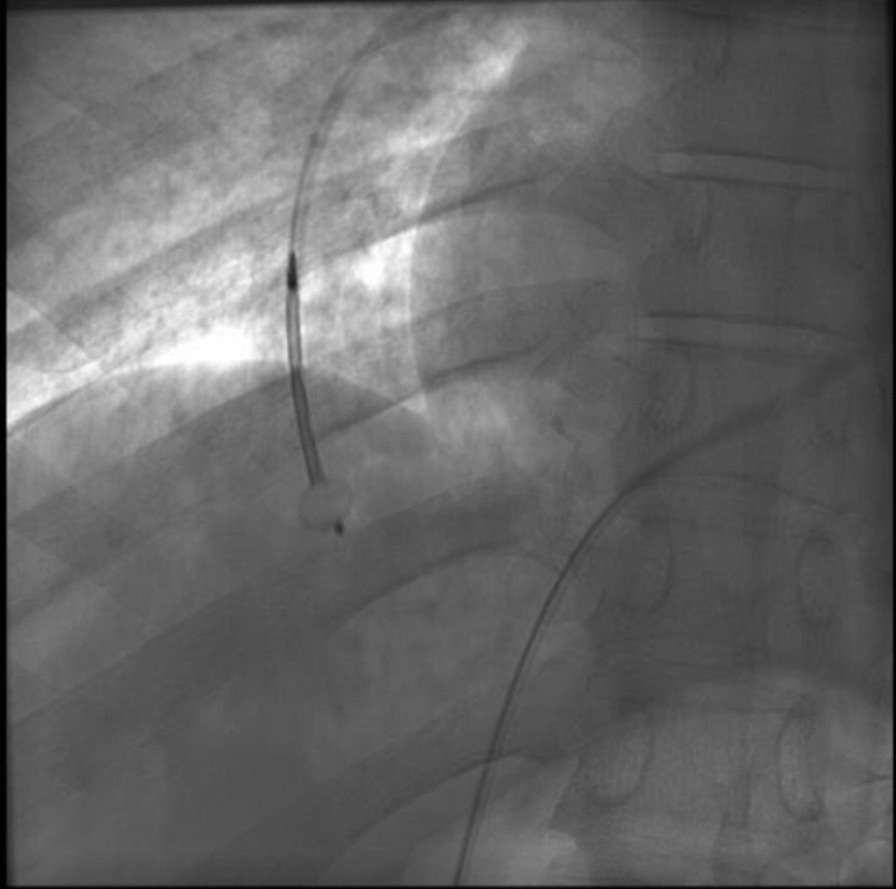
Computed tomography angiogram image pre-embolization of the vasculature feeding pulmonary arteriovenous malformation

**Fig. 2:**
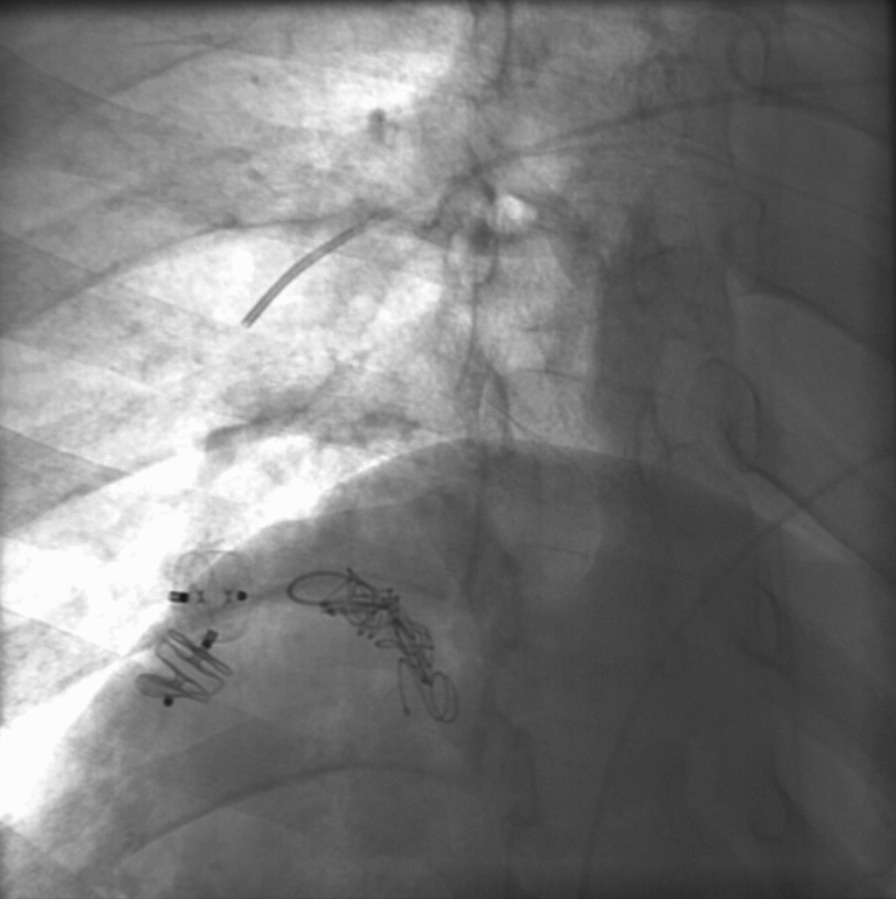
Computed tomography angiogram image showing successful embolization of vasculature feeding the pulmonary arteriovenous malformation using Amplatzer Vascular Plug II

Amplatzer Vascular Plug II was used for the embolization of two main vessels supplying the AVM, followed by five pushable coils being used for the embolization of another feeding arterial vessel (for pre- and post-embolization pictures, see Fig. 1 and 2, respectively). Post-procedure angiogram showed 70–80% embolization of the right pulmonary AVM, and no immediate post-procedure complications were noted. Percutaneous pulse oximetry saturation increased from 78% to 98% immediately on room air.

**Fig. 3:**
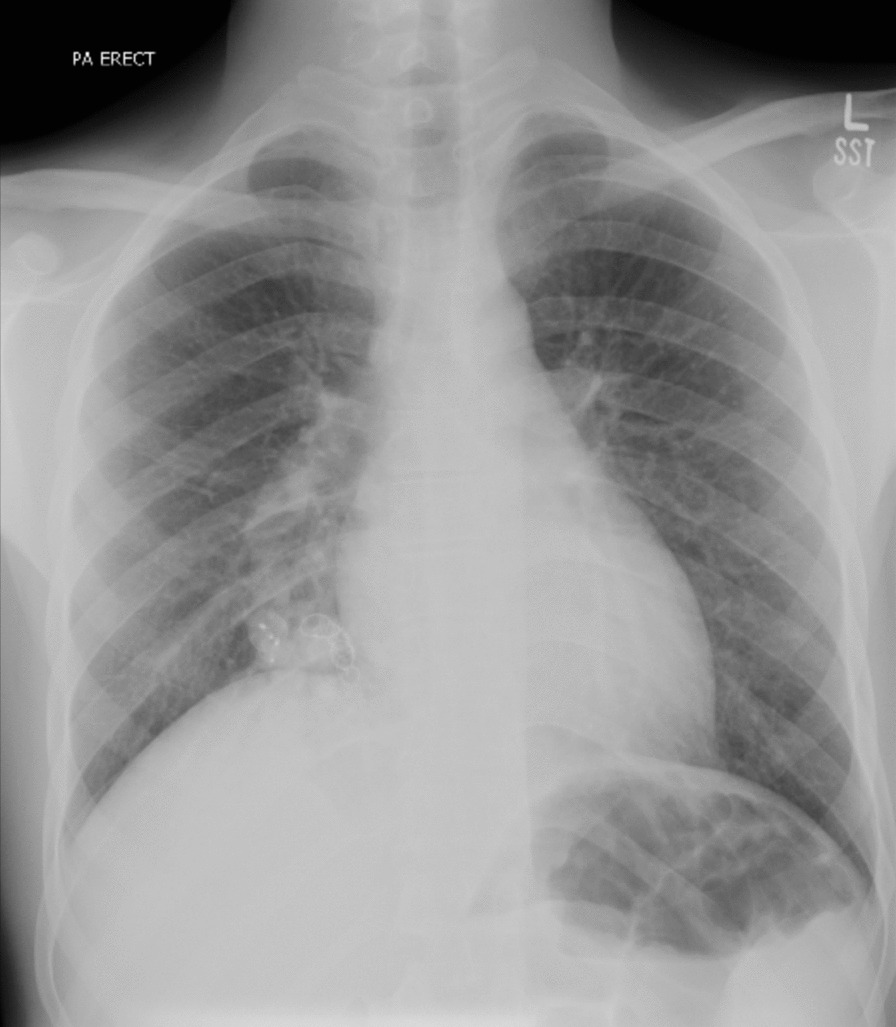
Chest X-ray at 1-week follow-up. Note the increased opacity around the cardiac silhouette at the site of angioembolization

At a follow-up visit 1 week post-procedure, O_2_ saturation was 87% on the pulse oximeter and he complained of a slight cough with hemoptysis, with subsequent chest X-ray appearing clear (Fig. 3). O_2_ saturation continued to rise on follow-up visits at 2 months (83% at rest, 77% on walking) and 8 months (90% at rest, 85% on walking). The patient was counseled regarding the need for supplemental oxygen and regular follow-ups; however, the patient refused supplemental oxygen, citing no breathing difficulties. The initial complaint of headaches had also resolved since the procedure on his monthly follow-up. Owing to patient request and financial considerations, no head imaging studies were performed for headaches. On follow-up 6 years later, the patient presented with worsening shortness of breath with a repeat CT angiogram showing recanalization of PAVM, thus requiring further embolization.

## Discussion

Pulmonary arteriovenous malformations are a relatively uncommon medical condition, affecting roughly 1 in every 2500 individuals. Of those suffering from PAVM, 80% have an underlying genetic condition, HHT, which has an estimated prevalence of 1 in 5000 [1–3]. HHT is an autosomal dominant condition, with more than 80% of the cases associated with a mutation in either the *ENG* (endoglin) gene, which results in HHT type 1, or the *ACVRL1* gene, which results in HHT type 2 [9]. Pulmonary AVMs are a more common manifestation of type 1 HHT in comparison with type 2 (with the prevalence of AVMs being as high as 29.2% in case of the presence of susceptibility locus) [10].

The pathophysiology of the disease is complicated and not completely understood, but it involves modifications in the normal angiogenesis pattern, which leads to the origination of abnormal blood vessels. This leads to a tangle of blood vessels, often known as a nidus, being formed which is void of capillaries. Without any capillaries, there is direct communication between the high-pressure pulmonary artery and the low-pressure pulmonary vein, resulting in an extremely fragile state [11].

The most common presentations of a PAVM can include repeated episodes of hemoptysis and epistaxis. Arterial hypertension is another commonly associated feature of PAVMs. Since a right-to-left shunt is established between the two vessels, compromising the blood flow to the lungs for oxygenation, patients with PAVM can present with hypoxemia and low oxygen saturations, as seen in our patient [4]. Since most patients with PAVM have HHT, they may present with acute or chronic gastrointestinal (GI) bleeding, characteristic telangiectatic lesions on the tongue, nose, and fingers, as well as iron-deficiency anemia from all the blood loss [12, 13].

This patient presented primarily with complaints of progressive and persistent frontal headaches. Headaches in PAVMs have been previously attributed to right to left shunting leading to hypoxia [14]. An argument has also been made regarding the presence of trigger substances such as vasoactive chemicals (for example, serotonin) and microthrombi that cross over the AVM rather than getting trapped in pulmonary capillaries, leading to increased cerebral vascular instability and cerebrovascular ischemic events [15]. Alternatively, the presence of cerebral arteriovenous malformations (CAVMs) has been reported in non-HHT patients in conjunction with PAVMs, although they are very rare and present as headaches or dizziness initially [16]. The presence of a CAVM could not be ruled out in this patient as no brain imaging had been conducted. Other causes of cerebrovascular causes of headaches (vasculitis, stroke, and so on) are less likely since the patient presented with no neurological deficits or systemic symptoms.

The patient had no complaint of hemoptysis or epistaxis. Similarly, there was no history of HHT in the patient’s family, nor did his parents or siblings report experiencing any similar symptoms. This is an extremely significant finding as approximately 90% of patients with PAVM have underlying HHT, and large, complex PAVMs, as seen in our patients are extremely rare in non-HHT patients [17, 18]. Furthermore, although most patients with HHT and hence PAVM have a complaint of iron-deficiency anemia, our patient in contrast was suffering from polycythemia, with a high Hb count of 18 g/dl. This can be explained as a compensatory mechanism of the body to produce more red blood cells (RBCs) in hypoxic conditions, with low O_2_ partial pressures. While polycythemia can be attributed to a multitude of physiological and pathological factors, intensive blood workup was avoided owing to financial considerations. Another explanation, although less likely, could be high-altitude polycythemia as the patient was a native resident living at high altitude (> 2000 m), however, no previous bloodwork was available to confirm this. Moreover, on the follow-up visit after successful embolization, the Hb of the patient dropped to 16.1 (within the normal range), which suggests that polycythemia was in fact due to PAVM and subsequent secondary hypoxemia.

PAVMs are usually diagnosed on the basis of clinical suspicion followed by imaging. Usually, a CT angiogram of the chest is the preferred modality; however, transthoracic contrast echocardiography (TTE) is used as a tool for screening and defining the shunt. Other screening tests include pulse oximetry arterial oxygen saturation (SaO_2_), chest radiograph, arterial oxygen measurement (PaO_2_) at room air, and PaO_2_ while breathing 100% oxygen [5, 19]. The patient was diagnosed based on only a CT scan, and he underwent a right pulmonary artery angiogram to characterize the AVM.

PAVMs are commonly located in the lower and middle lobes as well as the lingula and based on treatment are divided into simple, complex, and diffuse. A simple PAVM is fed by a single pulmonary segmental artery; complex PAVMs have multiple feeders. Diffuse PAVMs are rare (5%) and involve a lung lobe’s entire subsegmental/segmental artery [6, 7]. The efferent vessel drains into branches of the pulmonary vein. In this case, the lesion is present in the right lower lobe with multiple feeding arteries supplied by the right main pulmonary artery and drainage by the right inferior pulmonary vein. The lesion itself is made up of multiple tortuous vessels and large draining veins that are features of a complex AVM.

Treatment for PAVM can be surgical or endovascular, with the latter having more popularity. Surgical procedures such as pneumonectomy, lobectomy, and local excision are exclusively used for PAVMs that are unsuitable for embolization owing to a limited extent. Lung transplants have also been considered for diffuse PAVMs (especially in patients with HHT), though they have a poorer outcome. Endovascular techniques however are now considered first-line therapy for PAVM in all symptomatic and asymptomatic patients with PAVM > 2 mm. Transcatheter embolization is highly effective with high success rates and low incidence of complications (1%). These are carried out via vascular plugs [Amplatzer Vascular Plug (AVP) most commonly], detachable/pushable coils, and penumbra occlusion device (POD) as well as microvascular plugs for small PAVMs (feeders less than 3 mm in size). Simultaneously the symptoms such as hypoxemia, and increased hematocrit of PAVM are managed via oxygen supplementation and antiplatelet therapy in patients with a risk of cerebrovascular events [8, 20–24].

This patient underwent a right pulmonary artery angiogram and embolization of two of the feeding arteries using Amplatzer Vascular plug 2, while the third feeding artery was embolized by five pushable coils.

Mixing of deoxygenated blood through the PAVM leads to symptoms such as hypoxemia, fatigue, dyspnea, and cyanosis. It can further lead to complications such as paradoxical systemic embolism due to right to left shunting through the PAVM causing stroke, brain abscess, and seizure, especially with PAVM > 3 mm. Rupture of PAVM can also be seen in those AVM with thin walls, in pregnancy owing to hormonal changes that weaken the PAVM wall and increase circulating blood volume. Additionally, PAVMs are commonly located subpleurally, and thus ruptures can cause hemoptysis and hemothorax [25–28].

Post-procedural complications can also occur such as recanalization/regrowth of PAVMs or growth of new PAVMs as well as failed embolization and reperfusion of the aneurysmal sac, therefore contrast CT scan is recommended at 6 months and then 3–5 years post-procedure. As seen in our patient and reported in previous literature, recanalization can occur in almost 10% of cases 5–7 years post-procedure and can sometimes present even after decades [29]. Therefore, lifelong surveillance and counseling are important.

## Conclusion

Diffuse PAVMs, as seen in this patient, are rare, accounting for less than 5% of total PAVMs diagnosed. Moreover, this presentation in those without history of HHT is even rarer. Diagnosis and adequate management of pulmonary AVMs can be a challenge, especially in cases with an atypical presentation, such as in our patient with only persistent headaches as the primary complaint and the absence of usual symptoms such as dyspnea and hemoptysis. The progressive frontal headaches can be attributed to low oxygen saturation or the presence of a cerebral arteriovenous malformation (CAVM). Moreover, instead of the expected finding of anemia secondary to hemoptysis, our patient had polycythemia. Angioembolization provides a reliable and minimally invasive mode of management and should be used where available.

## Data Availability

Authors have full intellectual ownership of the article and agree to the data-sharing policy of the Journal.

## Abbreviations

PAVM: Pulmonary arteriovenous malformations
AVM: Arteriovenous malformations
CT: Computed tomography
CAVM: Cerebral arteriovenous malformations
HHT: Hereditary hemorrhagic telangiectasia
O_2_: Oxygen
TTE: Transthoracic contrast echocardiography
